# Minimally invasive approach associated with lower resource utilization after aortic and mitral valve surgery

**DOI:** 10.1016/j.xjon.2023.06.007

**Published:** 2023-06-28

**Authors:** NaYoung K. Yang, Fady K. Soliman, Russell J. Pepe, Nadia K. Palte, Jin Yoo, Sorasicha Nithikasem, Kayla N. Laraia, Abhishek Chakraborty, Joshua C. Chao, Gengo Sunagawa, Manabu Takebe, Anthony Lemaire, Hirohisa Ikegami, Mark J. Russo, Leonard Y. Lee

**Affiliations:** a Division of Cardiothoracic Surgery, Department of Surgery, Rutgers Robert Wood Johnson Medical School, New Brunswick, NJ; b Robert Wood Johnson University Hospital, New Brunswick, NJ

**Keywords:** minimally-invasive surgery, high-resource utilization, left heart valve surgery

## Abstract

**Objective:**

To investigate the effect of minimally invasive cardiac surgery (MICS) on resource utilization, cost, and postoperative outcomes in patients undergoing left-heart valve operations.

**Methods:**

Data were retrospectively reviewed for patients undergoing single-valve surgery (eg, aortic valve replacement, mitral valve replacement, or mitral valve repair) at a single center from 2018 to 2021, stratified by surgical approach: MICS vs full sternotomy (FS). Baseline characteristics and postoperative outcomes were compared. Primary outcome was high resource utilization, defined as direct procedure cost higher than the third quartile or either postoperative LOS ≥7 days or 30-day readmission. Secondary outcomes were direct cost, length of stay, 30-day readmission, in-hospital and 30-day mortality, and major morbidity. Multiple regression analysis was conducted, controlling for baseline characteristics, operative approach, valve operation, and lead surgeon to assess high resource utilization.

**Results:**

MICS was correlated with a significantly lower rate of high resource utilization (MICS, 31.25% [n = 115] vs FS 61.29% [n = 76]; *P* < .001). Median postoperative length of stay (MICS, 4 days [range, 3-6 days] vs FS, 6 days [range, 4 to 9 days]; *P* < .001) and direct cost (MICS, $22,900 [$19,500–$28,600] vs FS, $31,900 [$25,900–$50,000]; *P* < .001) were lower in the MICS group. FS patients were more likely to experience postoperative atrial fibrillation (*P* = .040) and renal failure (*P* = .027). Other outcomes did not differ between groups. Controlling for stratified Society of Thoracic Surgeons predicted risk of mortality, cardiac valve operation, and lead surgeon, FS demonstrated increased likelihood of high resource utilization (*P* < .001).

**Conclusions:**

MICS for left-heart valve pathology demonstrated improved postoperative outcomes and resource utilization.

## Graphical abstract

**Figure:**
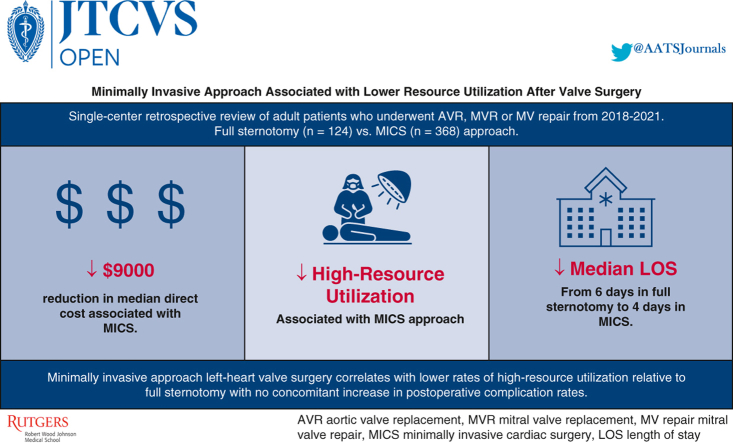

**Figure:**
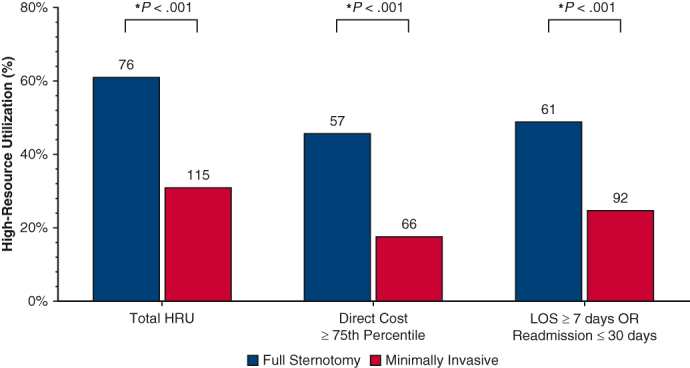
High-resource utilization overall and by definition stratified by operative approach.

**Central Message**

Minimally invasive approach in left-heart valve surgery correlates with lower resource utilization relative to full sternotomy with no concomitant increase in postoperative complication rates.

**Perspective**

Minimally invasive approaches are increasingly common across surgical specialties, with postoperative benefits and noninferior outcomes. MICS for left-heart valve pathology correlates with lower resource utilization relative to full sternotomy with no increase in postoperative complications. MICS may optimize resource allocation and provide higher value care.

See Discussion on page 81.

First revolutionized by British surgeons in 1986 through replacement of percutaneous nephrolithotomy with lithotripters,^1^^,^^2^ the minimally invasive approach has steadily been incorporated into many surgical fields.^2^ Nationwide, 3 million operations use a minimally invasive approach, with the case volume doubling in 2014 from 10 years prior.^3^ Within the field of cardiothoracic surgery, studies have shown the benefits of minimally invasive cardiac surgery (MICS) over traditional open techniques, including shorter hospital lengths of stay (LOS), decreased postoperative complications, including sternal infections and postoperative respiratory complications, and improved patient comfort with decreased postoperative pain.^4^

To quantify the benefits of MICS relative to traditional approaches, clinicians have determined measures based on procedure costs and outcomes. High-resource utilization (HRU)—defined as direct procedure cost higher than the third quartile^5^ or either postoperative LOS ≥7 days or 30-day readmission^6^, ^7^, ^8^, ^9^, ^10^—is 1 such metric that can be used to gauge the utility of MICS. Other metrics include nonhome discharge location.^10^

With thoughtful effort to curtail unnecessary surgical injury, minimally invasive techniques have gained favor in cardiac surgery, eventually propelling the popularization of minimally invasive approaches for valve operations.^11^, ^12^, ^13^ Although studies on postoperative outcomes have been conducted on isolated valve surgeries,^14^, ^15^, ^16^, ^17^, ^18^ minimally invasive valve operations have not been compared to open approaches comprehensively to include various valve types within 1 investigation. Furthermore, minimally invasive approaches to valve operations have not been compared to their full sternotomy (FS) counterparts in the context of patient resource utilization. Therefore, this study aims to investigate the effect of MICS on resource utilization, cost, and other postoperative outcomes in patients undergoing left heart valve operations.

## Methods

Patients aged 18 years or older who had undergone aortic valve replacement (AVR), mitral valve replacement (MVR), or mitral valve repair (MV repair) at a single tertiary care academic medical center between 2018 and 2021 were identified.

Data for this study was sourced from the cardiac surgery database of the academic center (Robert Wood Johnson University Hospital), and was developed according to The Society of Thoracic Surgeons (STS) Adult Cardiac Database version 4.20.1 definitions to include patient demographics, baseline clinical and perioperative characteristics, in-hospital outcomes, and 30-day outcomes. This study was approved by the Institutional Review Board of Robert Wood Johnson University Hospital under protocol #Pro2021001533 on September 21, 2021. Patients provided informed written consent for the publication of the study data.

### Patient Population

Surgeries included patients who underwent isolated AVR, MVR, or MV repair. Operative approach included either FS, partial sternotomy, or right minithoracotomy, with the minimally invasive cohort comprising operations with partial sternotomy and right minithoracotomy approaches. Selection for minimally invasive valve surgery vs conventional FS is based on shared decision making between the surgeon and the patient. Patients were stratified by operation approach (MICS vs FS). Patients who underwent transcatheter operations, underwent concomitant operations, had prior cardiac surgery of any kind, had indication for endocarditis, or who underwent an emergency operation were excluded from the study.

### Statistical Analyses

Baseline patient demographics, along with clinical and perioperative characteristics, were evaluated and compared. Primary outcome was HRU, defined as patients with either direct cost higher than the third quartile^5^ and/or either postoperative LOS ≥7 days or 30-day readmission.^6^, ^7^, ^8^, ^9^, ^10^ Secondary outcomes investigated were total direct costs—defined as expenses directly related to patient care, such as procedure and periprocedural costs^19^—postoperative LOS, 30-day readmission, in-hospital and 30-day mortality, along with other postoperative complications including atrial fibrillation, acute kidney injury, bleeding requiring reoperation, hospital readmission, reintubation, and stroke. Intraoperative bypass and crossclamp times, along with intraoperative and postoperative blood product utilization were also evaluated as secondary outcomes.

Outcomes of continuous and categorical variables are reported as medians and interquartile ranges (IQR) (25th–75th percentiles) or frequencies and proportions (%), respectively. Wilcoxon rank sum test and Fisher exact tests were used for comparison between cohorts.

For further analysis, a multivariable regression analysis was performed, controlling for operative approach (either FS or MICS), valve operation, lead surgeon, and STS predicted risk of mortality (STS PROM) score. Originally a continuous variable, “STS PROM score was stratified by low, medium, and high risk defined as 1% to 4%, 4% to 8%, and ≥8% risk, respectively, for this regression analysis. Thresholds for stratification of STS PROM scores were based on prior literature.^20^ Controlling for the STS risk score ultimately controlled for baseline characteristics intrinsically included in calculating the patient's score, including factors of age, gender, race, ethnicity, preoperative atrial fibrillation, body mass index, congestive heart failure, prior stroke or cerebrovascular disease, chronic renal failure requiring dialysis, smoking history/status, diabetes, preoperative ejection fraction, hypertension, hyperlipidemia, and New York Heart Association (NYHA) functional class. Statistical analysis was conducted using statistical program JMP Statistical Discovery Pro 16.2.0 (SAS Institute Inc).

## Results

The academic center has 28 operating rooms, with an average annual surgical volume of ∼15,000 cases. Approximately 10% of these are cardiac surgeries, with 1400 open and 350 transcatheter valve operations performed annually. Notably, 650 of these are valve procedures. Of 492 patients included in the study, 368 (74.80%) underwent MICS and 124 (25.20%) underwent FS. All patients, irrespective of surgical approach, utilized a multimodal Enhanced Recovery After Surgery (ERAS) protocol that included nutritional supplementation, prehabilitation, goal-directed therapy, multimodal opioid-sparing pain management, bowel motility prophylaxis, early mobilization, and multimodal analgesia. Of those who underwent MICS, 94.56% (n = 348) had right minithoracotomies (∼4 cm anterior incision, just right lateral of the sternum in the second intercostal space) and the remaining 4.44% underwent partial sternotomies (∼6 cm incision with a sternotomy “T'd” into the third or fourth intercostal space).

Baseline demographic and clinical characteristics of patients included are presented in Table 1. Patients who underwent minimally invasive surgery were of older age (*P* = .014) and were more likely to be women (*P* = .004). Whereas patients in the FS cohort had a greater proportion of those identifying with Hispanic ethnicity (*P* = .013), race did not differ significantly between the 2 cohorts overall (*P* = .265). The proportion of patients with diabetes mellitus (*P* = .182) or hypertension (*P* = .395) did not differ between the two cohorts. NYHA functional class >III did not differ between the two cohorts (*P* = .258), along with STS PROM score (*P* = .733).

**Table 1:** Baseline characteristics of minimally invasive cardiac surgery and full sternotomy patients by procedure type∗

Characteristic	Surgical approach		*P* value ⍺ = 0.05
	Full sternotomy (n = 124)	Minimally-invasive surgery (n = 368)	
Baseline demographics			
Age (y)	63 (55-70)	66 (58-73)	**0.014**
Female	37 (29.84)	165 (44.84)	**0.004**
Hispanic ethnicity	18 (14.52)	28 (7.61)	**0.013**
Race			0.265
White	61 (49.19)	211 (57.65)	
Black	11 (8.87)	17 (4.64)	
Asian	5 (4.03)	16 (4.37)	
Other (eg, Hispanic ethnicity)	44 (35.48)	106 (28.96)	
Clinical characteristics			
Atrial fibrillation	14 (11.48)	77 (21.15)	**0.018**
Body mass index	27.98 (24.28-33.23)	27.68 (24.38-31.63)	0.452
Congestive heart failure	39 (31.45)	94 (25.54)	0.200
Prior CVA	2 (1.63)	14 (5.34)	0.088
Cerebrovascular disease	17 (13.71)	40 (10.87)	0.153
Chronic lung disease ≥ moderate	21 (16.91)	28 (7.61)	**0.003**
Chronic renal failure	2 (1.61)	7 (1.90)	0.835
Cigarette smoking			0.734
History	41 (33.06)	113 (30.71)	
Current at time of surgery	14 (11.29)	32 (8.70)	
Diabetes mellitus	32 (25.81)	74 (20.11)	0.182
Ejection fraction (%)	58 (55-63)	60 (55-63)	0.639
Hypertension n (%)	98 (79.03)	277 (75.27)	0.395
Hypercholesterolemia n (%)	38 (30.89)	179 (49.18)	**<0.001**
NYHA functional class ≥III	25 (20.16)	58 (15.76)	0.258
STS PROM score (%)	1.36 (0.7-2.99)	1.32 (0.87-2.25)	0.733

Values are presented as median (interquartile range) or n (%). *CVA*, Cerebrovascular accident; *NYHA*, New York Heart Association; *STS PROM*, Society of Thoracic Surgeons predictive risk of mortality.

∗ All bold font indicates statistically significant difference at a *P*-value of 0.05.

Perioperative characteristics stratified by operative approach are described in Table 2. Cardiopulmonary bypass time (FS median, 106 minutes; interquartile range [IQR], 85-141.75 minutes vs MICS median, 65 minutes; IQR, 56-95 minutes; *P* < .001) and crossclamp time (FS median, 83 minutes; IQR, 54.25-112.25 minutes vs MICS median, 46 days; IQR, 39-65 minutes; *P* < .001) were significantly longer with FS approach operation. Intensive care unit (ICU) LOS (FS median, 28.5 days; IQR, 21-51.75 days vs MICS median, 15; IQR, 8-27 days; *P* < .001) was also longer in patients who underwent FS operative approach. Patients undergoing MICS were more likely to undergo on-table extubation (*P* < .001) and less likely to receive intraoperative and postoperative transfusions (*P* < .001).

**Table 2:** Perioperative outcomes of patients undergoing minimally invasive cardiac surgery and full sternotomy by procedure type∗

Characteristic	Surgical approach		*P* value ⍺ = 0.05
	Full sternotomy (n = 124)	Minimally-invasive surgery (n = 368)	
Perioperative			
Bypass time (min)	106 (85-141.75)	65 (56-95)	**<0.001**
Crossclamp time (min)	83 (54.25-112.25)	46 (39-67)	**<0.001**
On-table extubation	12 (9.68)	183 (49.73)	**<0.001**
ICU LOS (h)	28.5 (21-51.75)	15 (8-27)	**<0.001**
Operating surgeon			
Surgeon A	39 (8.01)	258 (52.98)	**<0.001**
Surgeon B	27 (5.54)	52 (10.68)	
Surgeon C	10 (2.05)	38 (7.80)	
Surgeon D	36 (7.39)	0 (0.00)	
Surgeon E	10 (2.05)	17 (3.49)	
Valve operation			
Aortic valve replacement	96 (19.51)	196 (39.84)	**<0.001**
Mitral valve replacement	25 (5.08)	54 (10.98)	
Mitral valve repair	3 (0.61)	118 (23.98)	
Blood products, ≥2 U			
Intraoperative			
Packed RBCs	20 (16.13)	7 (1.90)	**<0.001**
Fresh frozen plasma	28 (22.58)	11 (2.99)	**<0.001**
Cryoprecipitate	13 (10.48)	7 (1.90)	**<0.001**
Platelets	48 (38.71)	22 (5.98)	**<0.001**
Postoperative			
Packed RBCs	35 (28.23)	25 (6.79)	**<0.001**
Fresh frozen plasma	26 (20.97)	37 (10.05)	**0.002**
Cryoprecipitate	16 (12.90)	7 (1.90)	**<0.001**
Platelets	19 (15.32)	12 (3.26)	**<0.001**

values are presented as median (interquartile range) or n (%). *ICU LOS*, Intensive care unit length of stay; *RBCs*, red blood cells.

∗ All bold font indicates statistically significant difference at a *P*-value of 0.05.

**Figure 1:**
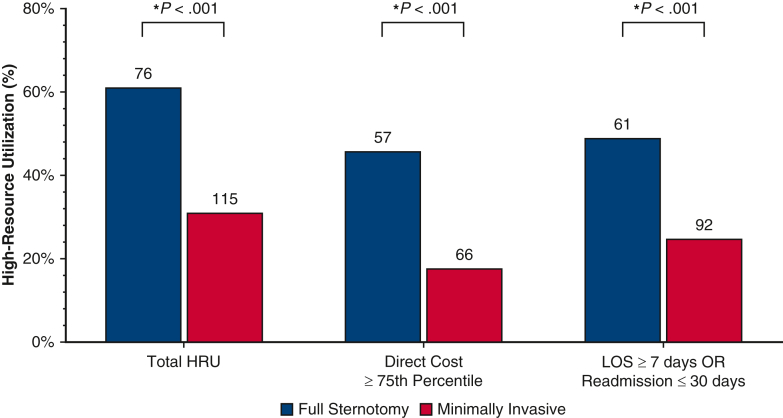
Comparison of high-resource utilization overall and separated by definition: direct procedure cost higher than the third quartile or either postoperative length of stay (*LOS*) ≥7 days or 30-day readmission between patients who underwent valve operations stratified by operative approach (full sternotomy vs minimally invasive surgery). By both separate definitions and composite high resource utilization, we find that patients who underwent full sternotomy cardiac valve operations experienced significantly higher rates of high resource utilization. *HRU*, High resource utilization; *OR*, odds ratio.

The MICS group contained a significantly lower proportion of patients in the HRU category: the MICS group underwent fewer operations with a direct cost higher than the 75th percentile ($33,000) compared with the FS group (17.93% [n = 66] vs 45.97% [n = 57]; *P* < .001). The MICS group contained fewer patients with either a postoperative LOS >7 days and/or readmission within 30 days, compared with the FS group (25.0% [n = 92] vs 49.19% [n = 61]; *P* < .001) (Figure 1).

**Figure 2:**
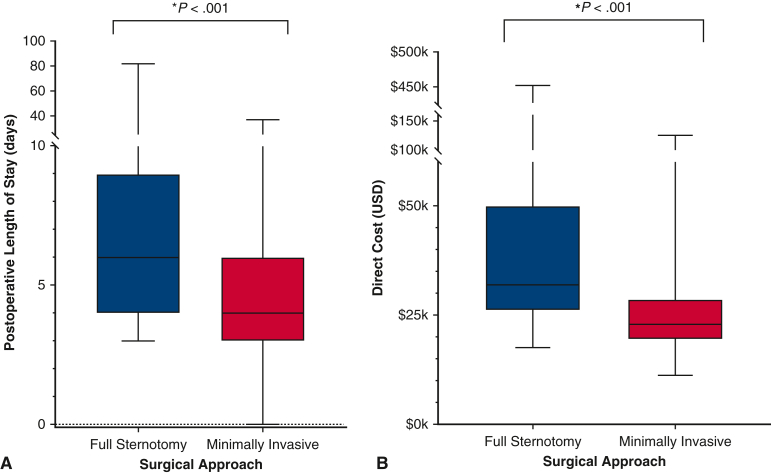
Comparison of (A) postoperative length of stay, and (B) total direct cost of patients who underwent valve operations stratified by operative approach. Patients who underwent full sternotomy experienced both significantly longer postoperative length of stay and higher direct costs.

**Figure 3:**
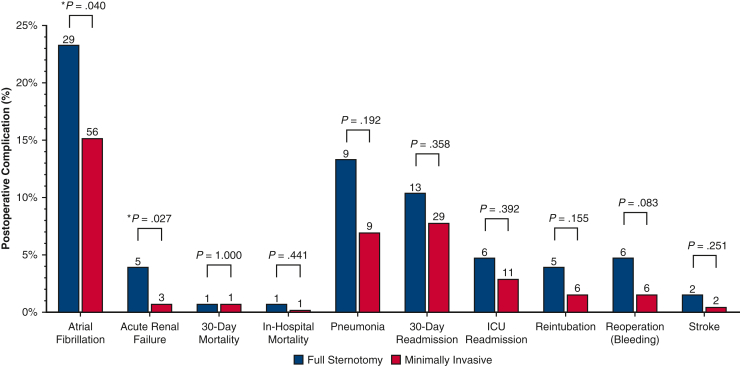
Postoperative complications, mortality, and readmission rates after left-heart valve surgery in patient cohorts stratified by operative approach. No significant difference was observed in mortality, readmission, and postoperative complications. Patients who underwent the full sternotomy approach were more likely to experience postoperative atrial fibrillation and renal failure. *ICU*, Intensive care unit.

In the total population, there were 4 (0.81%) mortalities; postoperative LOS ranged from 3 to 6.25 days (IQR) and total direct cost from $20.3,000 to $32.9k (IQR). Postoperative LOS (MICS median, 4 days; IQR, 3-6 days vs FS, 6 days; IQR, 4-9 days; *P* < .001) and direct costs (MICS median, $22,900; IQR, $19,500–$28,600] vs FS median, $31,900; IQR, $25,900–$50,000; *P* < .001) were lower in operations with a minimally-invasive approach (Figure 2). Postoperative 30-day mortality did not differ between patient cohorts even when contextualized by in-hospital mortality, ICU readmission, and 30-day readmission rates, which also did not show differences between the 2 cohorts. Other postoperative complications such as bleeding requiring reoperation, reintubation, stroke, and pneumonia showed no significant difference between the MICS and sternotomy groups. However, patients who underwent FS approach were more likely to experience postoperative atrial fibrillation (*P* = .040) and renal failure (*P* = .027) (Figure 3).

**Figure 4:**
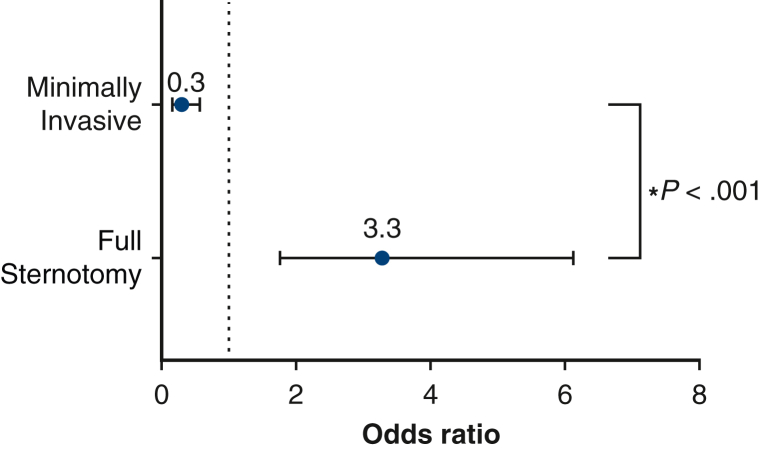
Forest plot of nominal logistic regression depicting full sternotomy (FS) vs minimally invasive cardiac surgery (MICS) and their respective odds of patients experiencing high resource utilization (HRU) controlled for lead surgeon, operation (aortic valve replacement [AVR], mitral valve replacement [MVR], or mitral valve [MV] repair), and Society of Thoracic Surgeons predicted risk of mortality score stratified into low, medium, and high risk defined as 1% to 4%, 4% to 8%, and ≥8% risk, respectively. The model showed that patients who underwent FS had 3.28 times the odds of HRU compared with their counterparts undergoing MICS (FS OR, 3.28; 95% CI, 1.76-6.13 vs MICS OR, 0.30; 95% CI, 0.16-0.57; *P* < .001).

Nominal logistic fit for HRU controlling for stratified STS risk (low, medium, and high), type of cardiac valve operation (AVR, MVR, MV repair), and lead surgeon showed a significant model (*P* < .001), in with patients who underwent FS significantly higher odds of high resource utilization compared with their minimally invasive counterparts (FS odds ratio [OR], 3.28; 95% CI, 1.76-6.13 vs MICS OR, 0.30; 95% CI, 0.16-0.57; *P* < .001) (Figure 4). Furthermore, the lead surgeon had a significant effect on odds of HRU, with more experienced surgeons showing decreased odds of HRU compared with those with fewer years of experience.

## Discussion

This study compared minimally invasive vs FS approaches for AVR, MVR, or MV repair at a single academic medical center. Our results indicate that minimally invasive approach is associated with shorter cardiopulmonary bypass and crossclamp times, shorter ICU LOS, fewer transfusion requirements, and lower postoperative rates of acute kidney injury and atrial fibrillation. Additionally, the MICS approach to left-heart valve surgery is associated with shorter LOS, lower cost after valve surgery, and ultimately lower rates of HRU, even when controlled for STS PROM, valve operation, and lead surgeon. Minimally invasive approaches did not compromise clinical outcomes as evidenced by the lack of differences in postoperative complications.

### Clinical Outcomes of Minimally Invasive Valve Surgery

Findings in this study build on the growing literature in favor of minimally invasive valve surgery. A propensity score-matched cohort analysis conducted by Bowdish and colleagues^21^ reported no significant difference in mortality (2.5% vs 1.0%; *P* = .28), and postoperative complication rates with shorter ICU LOS and transfusion requirements after traditional FS vs right mini-thoracotomy AVR. Numerous other studies have replicated these outcomes at other high volume centers utilizing minimally invasive approaches.^22^, ^23^, ^24^ These previous findings reported in the literature are similar to the mortality effect observed in our current study, demonstrating the safety of minimally invasive techniques employed at high-volume centers with experienced cardiac surgery teams.

Despite the evidence reporting benefits of MICS, barriers remain precluding its wide-spread adoption. In a recent retrospective report published by Nissen and Nguyen,^25^ the authors identified a number of factors essential to the success of a nascent MICS program. In addition to the logistical demands required to assemble and maintain a high-complexity team, MICS programs require unique administrative support and specialized education and training for practitioners. Additionally, adequate referral volume is required to allow for patient safety measures to become part of routine care while maintaining appropriate patient selection and increase surgeon chances of better outcomes through increased practice and experience.^26^ The marketing strategy for a MICS program, therefore, must provide and advertise elements of patient experience, recruitment of specialized health care professionals, and availability of cutting-edge equipment. The academic center in this study conducts ∼100 MICS prcedures per year, with operations conducted under experienced surgeons who, contrary to existing literature citing longer bypass and crossclamp times,^27^ are able to achieve shorter times in both areas. This is most likely possible through the extensive experience by both the surgeon and operating room team that over time have cultivated the expertise to achieve these shorter bypass and crossclamp times.

### Resource Utilization and Cost of Minimally Invasive Valve Surgery

In addition to the clinical outcomes of MICS suggesting substantial benefit over traditional FS, the effect on healthcare economics and resource utilization provide another strong argument for its wide-spread adoption. This is especially important in the setting of a health care system that is considered among the highest consumers of gross domestic product expenditure per capita in the world.^28^ Our study demonstrates a median direct cost difference of $9000 between full sternotomy ($31,900) vs minimally invasive ($22,900) valve surgery. More than ∼80,000 aortic valve surgeries and ∼40,000 mitral surgeries are currently performed every year.^29^^,^^30^ Taken together, these efforts to shift toward the widespread implementation of MICS may result in substantial reductions in national health care expenditure.

### Implications of HRU in Healthcare Expenditure

In 2015, the Lancet Commission on Global Surgery proposed 6 corresponding core surgical indicators to assess surgical, anaesthetic, and obstetric health care systems in their ability of preparedness, service delivery, and cost protection: geographic access to a Bellwether hospital within 2 hours, surgical, anaesthetic, and obstetric provider density, total operative volume, in-hospital postoperative mortality, impoverishing, and catastrophic cost burden.^31^ Impoverishing and catastrophic costs can be assessed by definition of HRU used in this study. Increased direct cost and postoperative length of stay as seen in our FS population increases the burden, especially financially, on health care systems. Given the relationship between HRU and increased costs, which are then passed onto the hospital, there is benefit to the health care system when costs are decreased through lower resource utilization. No difference in postoperative complications was observed between the 2 groups in our study, thereby suggesting that the MICS approach provides an avenue for safe, effective valve repair or replacement without unnecessarily increasing financial burden on health care payers.

Beyond reducing the direct burden of cost on the health care system, MICS has been shown to correlate with decreased postoperative LOS. Prolonged hospital stay not only carries the cost of facilities, staff, and providers needed for patient care, but also increased risk of complications, including avoidable nosocomial infections, iatrogenic injury, and mortality.^32^, ^33^, ^34^ Patients undergoing MICS are likely to have a shorter LOS in comparison to those undergoing full FS, leading to reduced use of hospital facilities and resources.

Although costs of health care are often not directly translated directly to a patient's hospital invoice, patients may still inadvertently experience increased costs due to HRU. As an example, patients with a high deductible plan or large required copayment, depending on insurance coverage, may be required to pay additional costs that may present an unexpected hardship. Accordingly, decreasing resource utilization through limiting costs can decrease the financial burden on health care systems, and by extension, to patients and their families.

### Limitations

Despite the benefits shown in this study, several limitations exist. Given that this study is retrospective with an observational design, bias is present due to the lack of randomization and a priori data field selection. Moreover, the data used in this study was sourced from a single institution, thereby subjecting results to differences in surgeon technique and experience. Notably, majority of minimally invasive operations at this institution were conducted by a couple surgeons with extensive training and experience in minimally invasive surgery. Furthermore, patients undergoing surgery at this institution underwent an ERAS protocol, which may not be used universally at other institutions. Accordingly, these limitations may restrict the generalizability of these results to other institutions. Although this introduces the possibility of a confounding variable into the study, the variability of ERAS was deemed to not be 1 that would introduce significant bias. Surgeons with extensive experience in MICS also conducted full sternotomies regularly. Prospective studies in larger cohorts are warranted.

## Conclusions

**Figure 5:**
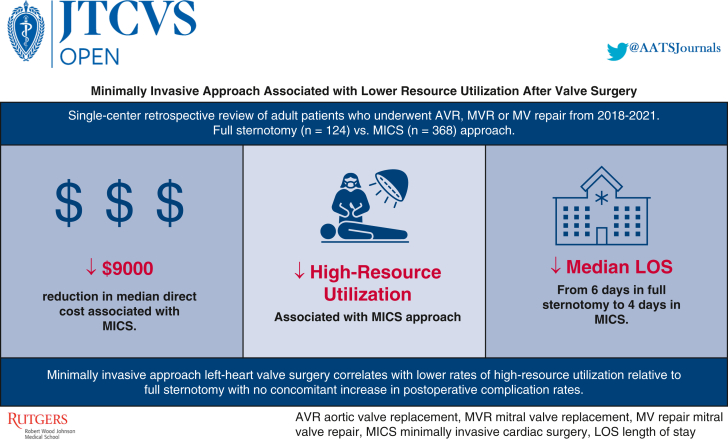
Outcomes of minimally invasive approach to left-heart valve surgery. Our results show that minimally invasive cardiac surgery (*MICS*) approach correlated with lower rates of high resource utilization, shorter length of stay (*LOS*), and lower direct cost relative to full sternotomy with no concomitant increase in postoperative complication rate. *AVR*, Aortic valve replacement; *MVR*, mitral valve replacement; *MV*, mitral valve.

Incorporation of minimally invasive surgery in various surgical specialties has been shown to provide postoperative benefits, including shorter hospital LOS, decreased postoperative complications, and greater patient comfort in the path to recuperation. HRU is 1 metric to compare FS vs minimally invasive approaches to cardiac surgery. Our study of one academic institution shows that left-heart valve surgery via MICS approach correlated with lower rates of HRU and lower direct cost relative to full sternotomy with no concomitant increase in postoperative complication rate (Figure 5). MICS may optimize resource allocation and provide higher value care for patients.

### Webcast

You can watch a Webcast of this AATS meeting presentation by going to: https://www.aats.org/resources/minimally-invasive-approach-associated-with-lower-resource-utilization-and-lower-cost-after-valve-surgery.

**Figure:**
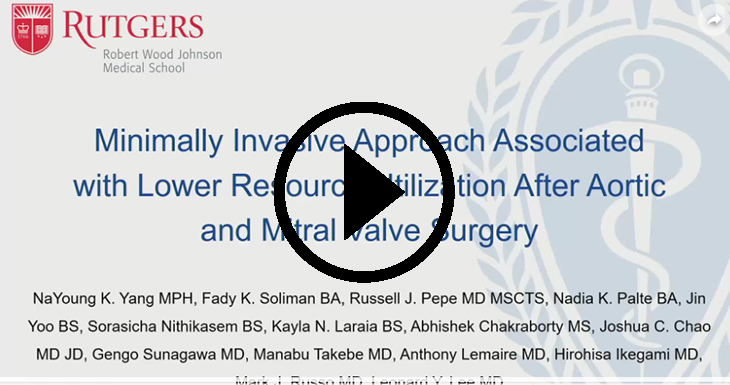

### Conflict of Interest Statement

Dr Russo discloses financial relationships with Edwards Lifesciences and Abbott Laboratories. Dr Lee discloses a financial relationship with Abbott Laboratories. All other authors reported no conflicts of interest.

The *Journal* policy requires editors and reviewers to disclose conflicts of interest and to decline handling or reviewing manuscripts for which they may have a conflict of interest. The editors and reviewers of this article have no conflicts of interest.
